# TMEFF2 Is a PDGF-AA Binding Protein with Methylation-Associated Gene Silencing in Multiple Cancer Types Including Glioma

**DOI:** 10.1371/journal.pone.0018608

**Published:** 2011-04-29

**Authors:** Kui Lin, James R. Taylor, Thomas D. Wu, Johnny Gutierrez, J. Michael Elliott, Jean-Michel Vernes, Hartmut Koeppen, Heidi S. Phillips, Frederic J. de Sauvage, Y. Gloria Meng

**Affiliations:** Genentech, South San Francisco, California, United States of America; Florida International University, United States of America

## Abstract

**Background:**

TMEFF2 is a protein containing a single EGF-like domain and two follistatin-like modules. The biological function of TMEFF2 remains unclear with conflicting reports suggesting both a positive and a negative association between TMEFF2 expression and human cancers.

**Methodology/Principal Findings:**

Here we report that the extracellular domain of TMEFF2 interacts with PDGF-AA. This interaction requires the amino terminal region of the extracellular domain containing the follistatin modules and cannot be mediated by the EGF-like domain alone. Furthermore, the extracellular domain of TMEFF2 interferes with PDGF-AA–stimulated fibroblast proliferation in a dose–dependent manner. TMEFF2 expression is downregulated in human brain cancers and is negatively correlated with PDGF-AA expression. Suppressed expression of TMEFF2 is associated with its hypermethylation in several human tumor types, including glioblastoma and cancers of ovarian, rectal, colon and lung origins. Analysis of glioma subtypes indicates that TMEFF2 hypermethylation and decreased expression are associated with a subset of non-Proneural gliomas that do not display CpG island methylator phentoype.

**Conclusions/Significance:**

These data provide the first evidence that TMEFF2 can function to regulate PDGF signaling and that it is hypermethylated and downregulated in glioma and several other cancers, thereby suggesting an important role for this protein in the etiology of human cancers.

## Introduction

TMEFF2, also known as tomoregulin [1], TPEF [2], HPP1 [3] and TENB2 [4], encodes a transmembrane protein that contains a single epidermal growth factor (EGF)-like domain and two follistatin-like modules [1], [4]–[6]. The biological function of TMEFF2 remains elusive with conflicting reports from different groups. Soluble forms of TMEFF2 extracellular domain have been reported to weakly stimulate erbB-4/HER4 tyrosine phosphorylation in MKN 28 gastric cancer cells [1], and promote survival of mesencephalic dopaminergic neurons in primary culture [6]. As evidence for its positive role in cell proliferation, elevated TMEFF2 expression has been associated with higher prostate cancer grade and hormone independence by several groups [4], [7], [8]. In contrast, others have reported down-regulation of TMEFF2 in androgen-independent prostate cancer xenografts, as well as growth inhibition induced by ectopic expression of TMEFF2 in androgen-independent prostate cancer cell lines [5]. Moreover, the 5′-region of TMEFF2 gene is frequently hypermethylated in some cancers [2], [3], [9]–[16], suggesting a possible tumor suppressor role of TMEFF2 in these cancers.

Platelet-derived growth factors (PDGFs) not only play important roles in developmental and physiological processes, but also are directly implicated in human cancer and other proliferative disorders (reviewed in [17] and [18]). The human genome contains four PDGF ligands, PDGF-A, B, C and D, and two receptors, PDGFRα and PDGFRβ All PDGFs can form functional disulfide-linked homodimers, while only PDGF-A and B have been shown to form functional heterodimers. PDGFRs also function as homo- and hetero-dimers that differ in their affinities to different PDGF dimers (reviewed in [17] and [18]). The α subunit of PDGFR has been shown to bind the PDGF-A, B and C chains, whereas the β subunit is believed to bind only the B and D chains. The biological responses induced by the different PDGF ligands depend on the relative numbers of the receptor subunits on a given cell type and the specific PDGF dimers present.

Follistatin module-containing proteins have been previously shown to be able to bind and modulate the function of a variety of growth factors including members of the transforming growth factor beta (TGF-β family, PDGFs, and vascular endothelial growth factor (VEGF) [19]–[24]. To date, however, no binding partner has been reported for TMEFF2. In this report, we have identified PDGF-AA as a growth factor that interacts with TMEFF2. Moreover, we show that the extracellular domain of TMEFF2 interferes with PDGF-AA–stimulated fibroblast proliferation in a dose–dependent manner. Our data provide the first evidence that TMEFF2 can function to regulate PDGF signaling, and give new mechanistic insights into the seemingly conflicting roles of TMEFF2 in human cancers. In addition, we show for the first time that the expression of TMEFF2 is downregulated in glioma and several other cancers and that this downregulation correlates with DNA methylation. Together these data suggest an important role of TMEFF2 in the development and progression of human cancers.

## Results

### The extracellular domain of TMEFF2 interacts with PDGF-AA

**Figure 1:**
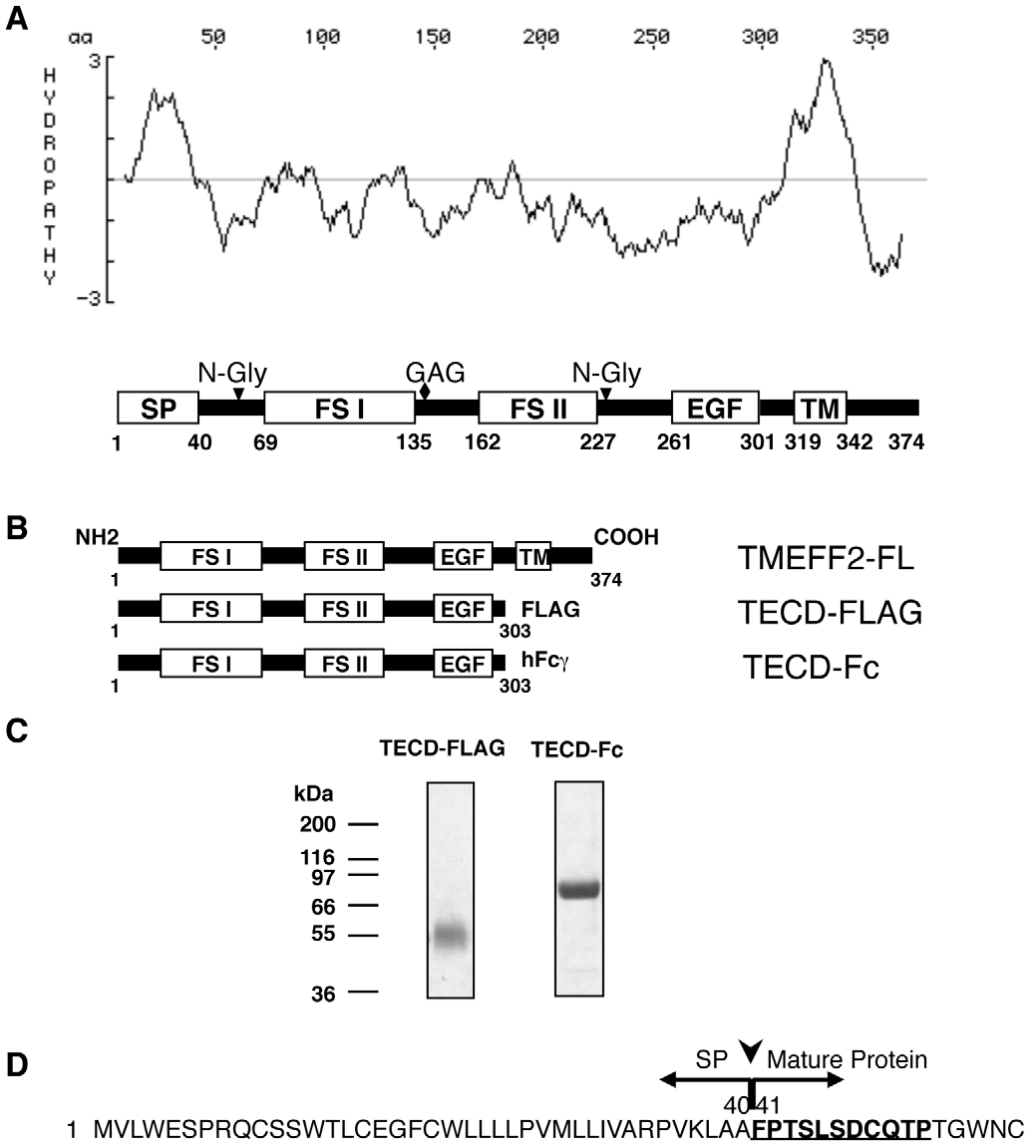
Expression and purification of recombinant ECD of TMEFF2. (**A**) Hydropathy plot of TMEFF2 protein based on the algorithm of Kyte and Doolittle [53] and the predicted domain structure based on NT sequencing of the recombinant TECD in this study and Horie et al., 2000 [6]. SP, signal peptide; FS I, follistatin-like domain I; FS II, follistatin-like domain II; EGF, epidermal growth factor-like domain; TM, transmembrane domain; N-Gly, potential sites for N-linked glycosylation; GAG, potential site of glycosaminoglycan attachment. (**B**) Schematic representation of the recombinant TECD-FLAG and TECD-Fc fusion proteins aligned with the full length TMEFF2 (TMEFF2-FL). (**C**) Purified TECD-FLAG and TECD-Fc were analyzed by SDS-PAGE under reducing conditions with Coomassie blue staining. (**D**) NT sequencing of the purified TECD-FLAG and TECD-Fc revealed the cleavage site of the signal peptide. The amino acid sequence identified by NT sequencing is underlined. Arrowhead indicates the signal peptide cleavage site.

TMEFF2 is predicted to contain a transmembrane (TM) domain with an amino terminal (NT) signal peptide sequence (SP) (Fig. 1A). Recombinant proteins containing the extracellular domain (ECD) of TMEFF2 fused to a FLAG tag (TECD-FLAG) or the Fc portion of the human immunoglobulin gamma (hFcγ (TECD-Fc) at the carboxy-terminus (CT) were expressed in mammalian cells and purified from cell culture supernatants (Fig. 1B). The purified TECD-FLAG and TECD-Fc ran at the predicted ∼55 kDa and ∼70 kDa on SDS PAGE under reducing conditions, respectively (Fig. 1c). NT sequencing of the purified proteins revealed that the signal peptide was cleaved between residues 40 and 41 in both recombinant proteins (Fig. 1D).

**Figure 2:**
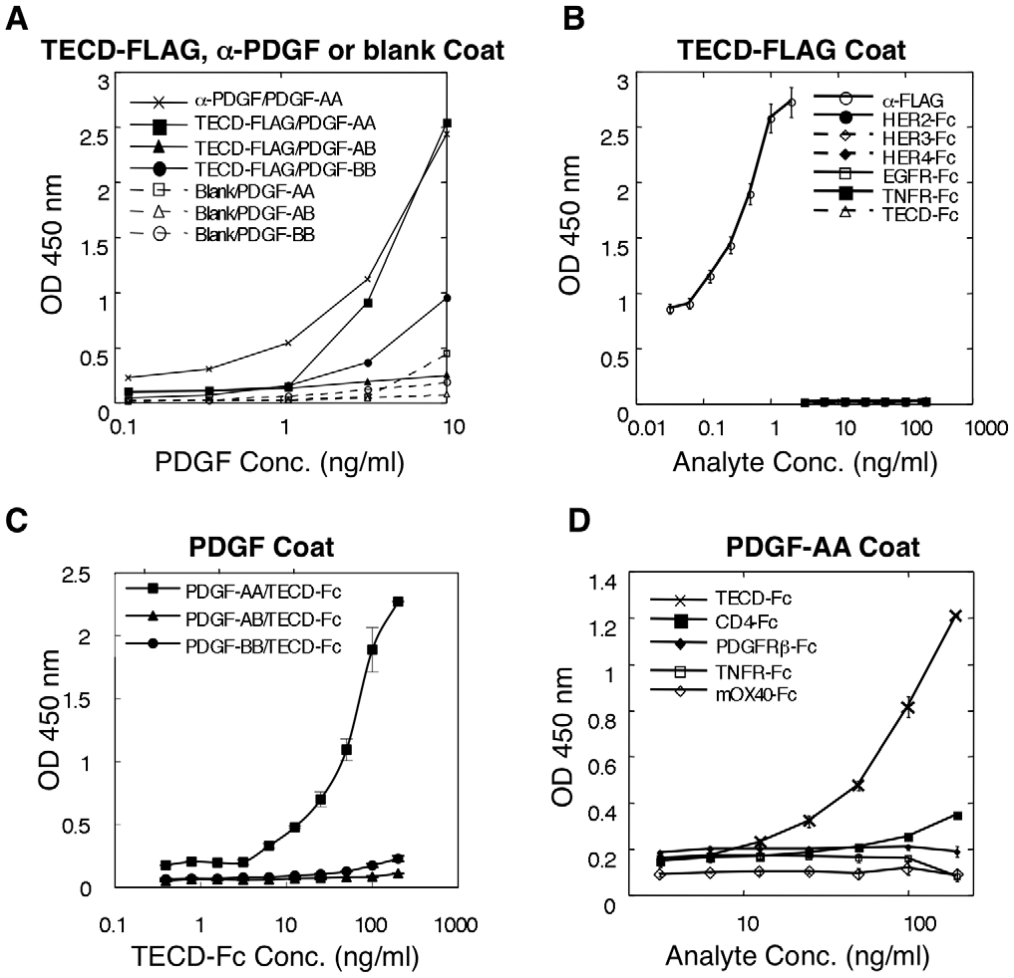
Binding of PDGF ligands and other recombinant proteins to immobilized TECD-FLAG (A,B) and binding of TECD-Fc to immobilized PDGF ligands (C,D). (**A**) Binding of dimeric PDGF ligands to TECD-FLAG coated wells. PDGF-AA, AB or BB were applied to TECD-FLAG coated wells (solid symbols) or blank wells (open symbols) and detected with biotinylated anti-PDGF-A (for PDGF-AA & AB) or PDGF-B (for PDGF-BB) antibodies followed by streptavidin-HRP. Anti-PDGF pAb coated wells were used as a positive control for PDGF-AA binding (x). (**B**) Binding of six recombinant Fc-tagged ECDs and an anti-FLAG mAb to TECD-FLAG coated wells. HRP-conjugated anti-mouse and anti-human Fcγ were used to detect anti-FLAG mAb and Fc-tagged proteins, respectively. (**C**) TECD-Fc was applied to wells coated with PDGF-AA, AB or BB and detected with HRP-conjugated anti-human Fcγ. (**D**) TECD-Fc and other Fc–tagged ECD of various transmembrane proteins were applied to PDGF-AA coated wells and detected with HRP-conjugated anti-human Fcγ. TNFR, tumor necrosis factor receptor; PDGFRβ, PDGF receptor β; mOX40, murine OX40. Error bars represent standard deviations between duplicates. Representative graphs of at least three independent experiments are shown.

Since follistatin (FS) module-containing proteins have been shown to interact with PDGF ligands [19], we examined the ability of each of the 3 dimeric forms of PDGF ligands, PDGF-AA, BB and AB, to interact with the ECD of TMEFF2 using Enzyme-Linked Immunosorbent Assays (ELISA). Using a biotinylated anti-PDGF-A antibody, we observed a dose-dependent binding when 1 to 10 ng/ml of PDGF-AA was added to the immobilized TECD-FLAG. A weak binding was detected using PDGF-BB and a biotinylated anti-PDGF-B antibody, whereas no significant binding was detected for PDGF-AB using the biotinylated anti-PDGF-A antibody (Fig. 2A). While there was only a slight background binding between PDGF-AA and the uncoated plastic wells, the binding of PDGF-AA to immobilized TECD-FLAG was comparable to its binding to an immobilized anti-PDGF antibody under the same conditions (Fig. 2A). No specific binding was detected for a variety of other proteins examined, including the EGF Receptor family members (EGFR, HER2, HER3 or HER4) and the tumor necrosis factor receptor (TNFR) fused to hFcγ. In addition, no significant binding was detected between the TMEFF2 ECDs themselves when TECD-Fc was used as an analyte. As a positive control, an anti-FLAG monoclonal antibody showed dose-dependent binding to the TECD-FLAG coated wells Fig. 2B).

To confirm that the binding observed is indeed due to the interaction between PDGF-AA and TMEFF2-ECD, we then immobilized the PDGF ligands on the plates, and applied the TMEFF2-ECD fused to a different tag, TECD-Fc, as an analyte. Consistent with the results obtained with immobilized TECD-FLAG, TECD-Fc exhibited significant dose-dependent binding only to immobilized PDGF-AA, but not AB, BB, CC or DD (Fig. 2C; supplemental Fig. S1A). Similar results were obtained using the label free ForteBio platform (Menlo Park, CA) to measure PDGF binding to biotinylated TMEFF2-FLAG immobilized on the streptavidin-coated sensor. PDGF-AA showed the strongest binding to TMEFF2 while PDGF-BB, AB, CC, and DD showed greatly reduced affinities (data not shown). A recombinant soluble PDGF receptor α extracellular domain (sRα), on the other hand, showed dose-dependent binding to all 3 immobilized PDGF dimers AA, AB and BB (supplemental Fig. S1B), whereas the PDGF receptor βECD-Fc (PDGFRβ-Fc) fusion protein was not able to bind PDGF-AA (Fig. 2D), consistent with the reported specificity of these receptors [25]–[27].

### TMEFF2 interacts with PDGF-AA through its FS module-containing region when expressed on the surface of mammalian cells

**Figure 3:**
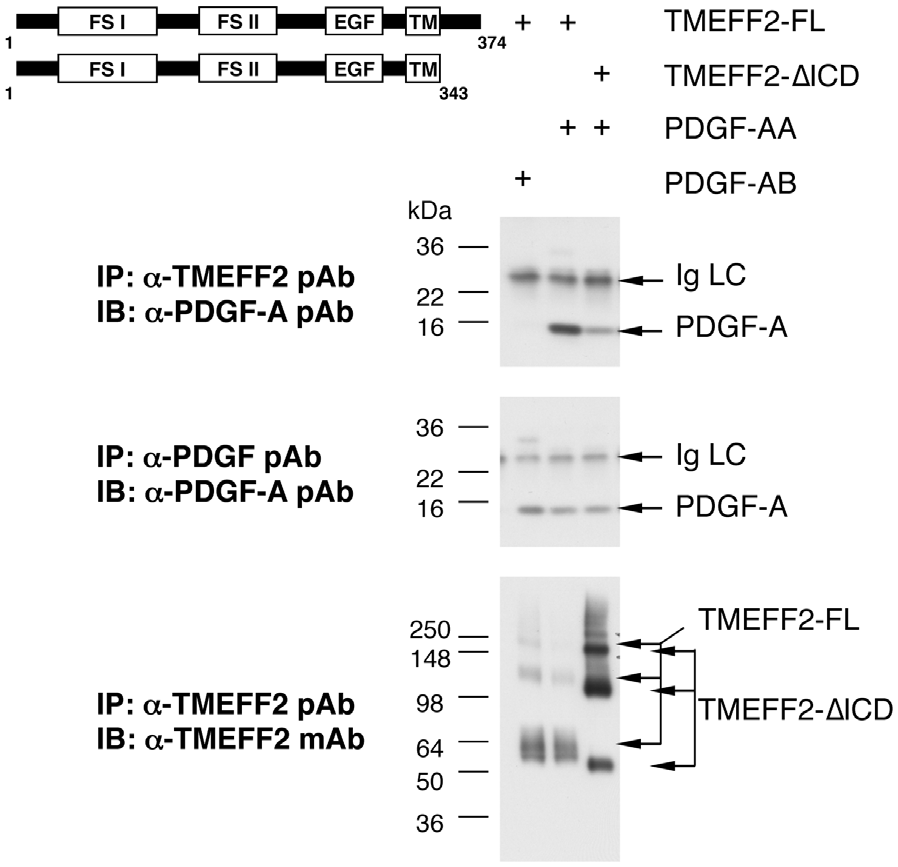
Co-immunoprecipitation of PDGF-AA with full-length or intracellular domain–truncated TMEFF2 expressed on the surface of 293 cells. Multiple bands of TMEFF2-FL and TMEFF2-ΔICD were detected by the anti-TMEFF2 mAb due to different degrees of glycosylation and proteoglycan attachment [4]. mAb, mouse monoclonal antibody; pAb, rabbit polyclonal antibody; Ig LC, light chain of the Ab used for the immunoprecipitation.

To determine if the ECD of TMEFF2 can interact with PDGF-AA when expressed on the surface of mammalian cells, we transfected 293 cells with constructs containing the full-length TMEFF2 (TMEFF2-FL), or a truncated TMEFF2 without the intracellular domain (TMEFF2-ΔICD) (Fig. 3). PDGF-AA or PDGF-AB was then added to the culture media and allowed to bind to the cell surface for 30 minutes. Unbound PDGF ligands were subsequently washed away and cell lysates were subjected to immunoprecipitation with either a polyclonal antibody (pAb) recognizing both PDGF-AA and AB dimers, or a pAb recognizing the ECD of TMEFF2. As shown in Fig. 3 & supplemental Fig. S8, an anti-PDGF-A antibody could detect the denatured PDGF-A monomer in the anti-PDGF immunoprecipitates from cells incubated with either PDGF-AA or PDGF-AB, suggesting that both PDGF dimers bound to the cell surface, either through interactions with specific receptors or extracellular matrix (ECM) proteins. However, PDGF-A was detected in the anti-TMEFF2 immunoprecipitates only from cells incubated with PDGF-AA but not from those incubated with PDGF-AB. In addition, PDGF-AA was present in anti-TMEFF2 immunoprecipitates from cells expressing either the full-length TMEFF2 or the ICD-truncated TMEFF2. This is consistent with the ELISA result showing that PDGF-AA but not PDGF-AB exhibited dose-dependent binding to the ECD of TMEFF2.

**Figure 4:**
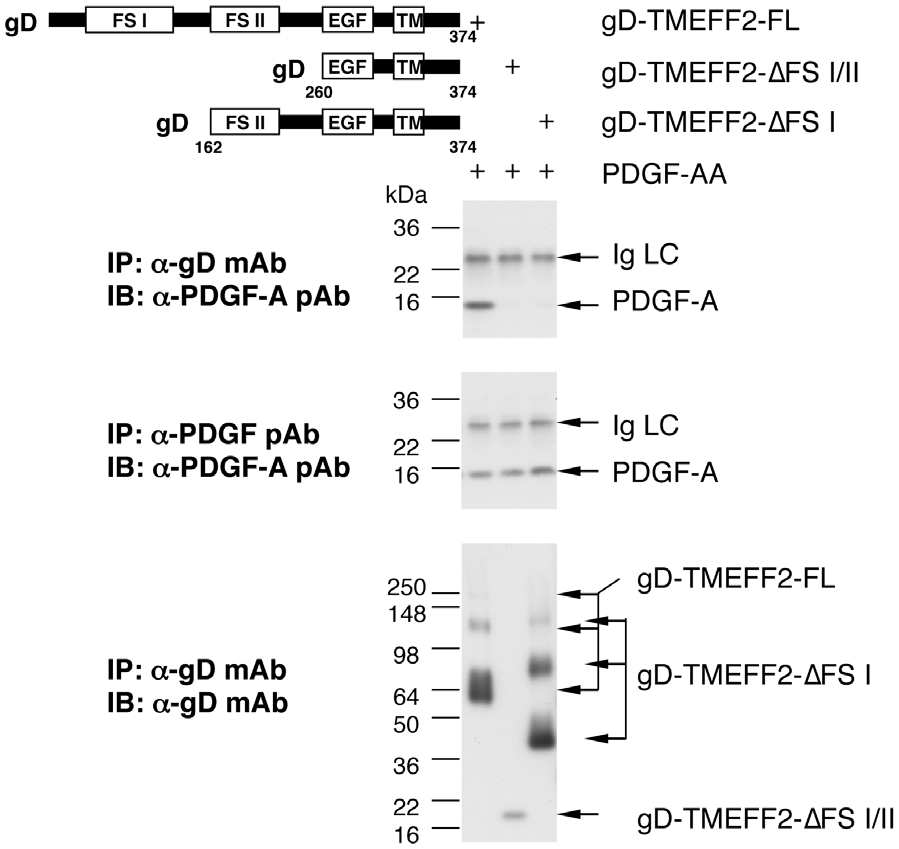
Interaction of PDGF-AA with gD-tagged deletion mutants of membrane–bound TMEFF2. Multiple bands of TMEFF2-FL and TMEFF2-ΔFS I were detected by the anti-TMEFF2 mAb due to different degrees of glycosylation and proteoglycan attachment [4].

TMEFF2 contains 2 FS modules and an EGF-like domain. To dissect which domains of TMEFF2 are involved in its interaction with PDGF-AA, we made Herpes simplex type 1 glycoprotein D (gD)-epitope tagged deletion mutants of TMEFF2 and examined their ability to bind PDGF-AA when expressed on the surface of 293 cells (Fig. 4 & supplemental Fig. S8). As expected, PDGF-AA co-immunoprecipitated with gD-tagged full-length TMEFF2 by an anti-gD monoclonal antibody. However, when gD-tagged TMEFF2 mutants lacking either the NT FS I (gD-TMEFF2-ΔFS I) or both of the FS modules (gD-TMEFF2-ΔFS I/II) were immunoprecipitated with the same anti-gD antibody, no PDGF-AA was brought down, although both mutant TMEFF2 proteins were brought down in the immunoprecipitates. FACS analysis also confirmed membrane expression of all 3 gD-tagged proteins (Supplemental Fig. S2). This suggests that NT regions containing the FS I domain are required for the PDGF-AA interaction, whereas EGF domain alone is insufficient for this interaction. Consistent with this result, a recombinant His-tagged tandem-array of the EGF domain of TMEFF2 also failed to show specific binding to PDGF-AA–coated plates by ELISA (data not shown).

### TMEFF2 modulates PDGF-stimulated proliferation of NR6 fibroblasts

PDGF ligands are potent mitogens of connective tissue cells, including fibroblasts, smooth muscle cells, chondrocytes, and some endothelial cells [17], [28], [29]. The finding that TMEFF2 interacts with PDGF-AA at ng/ml concentrations of both recombinant TMEFF2 ECD and PDGF-AA prompted us to examine the possibility that TMEFF2 may regulate PDGF-AA signaling. We first asked whether PDGFRα, the only receptor that binds PDGF-AA, could compete with TECD for PDGF-AA binding. As shown in Supplemental Fig. S1C, TECD-Fc binding to the PDGF-AA coated plate was blocked by the soluble extracellular domain of PDGFRα, sRα, in a dose dependent manner, indicating that TMEFF2 ECD and sRα bind to PDGF-AA at overlapping sites.

**Figure 5:**
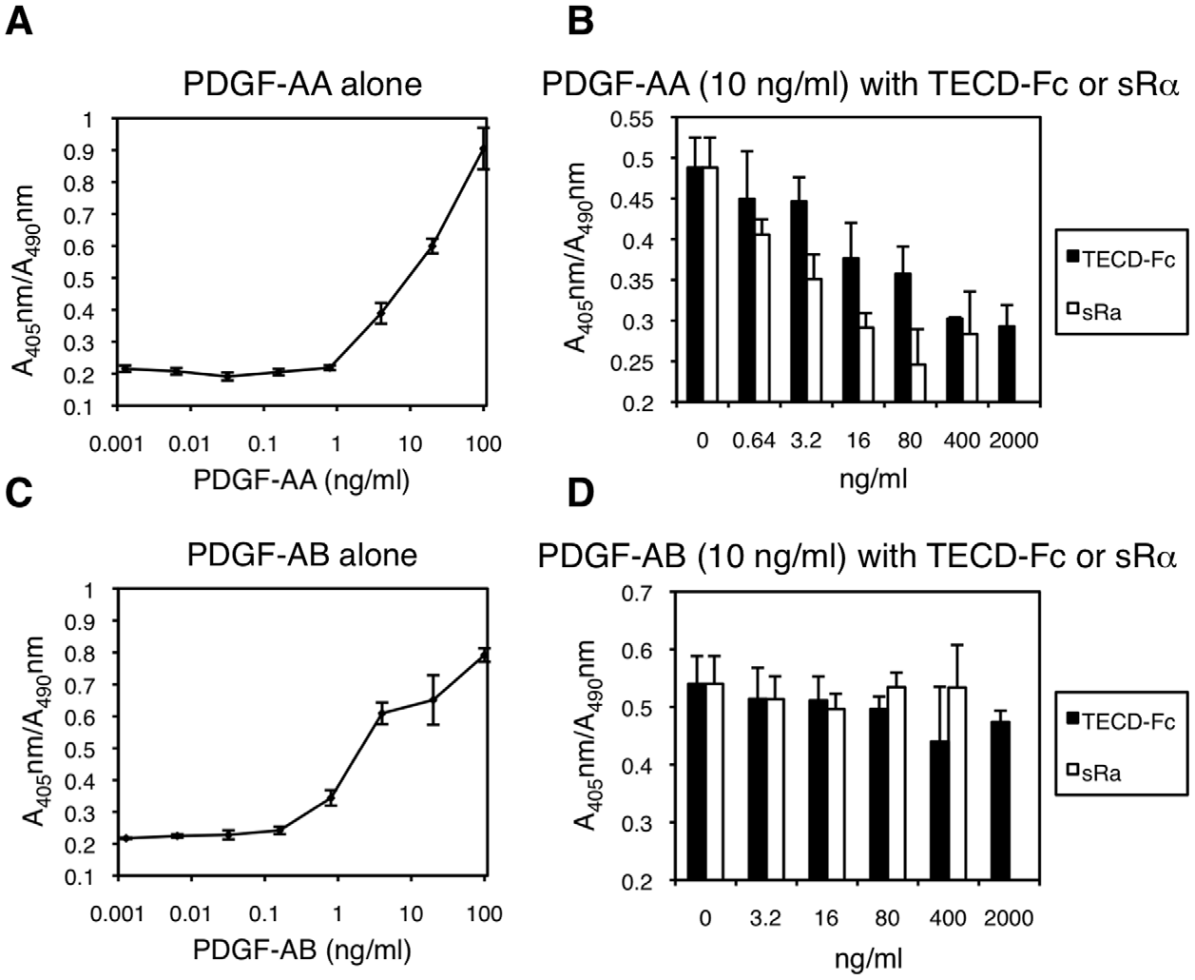
TECD-Fc interferes with PDGF-AA–stimulated proliferation of NR6 cells. (**A**) & (**C**) Dose-dependent stimulation of BrdU incorporation by PDGF-AA and PDGF-AB in NR6 cells. (**B**) & (**D**) Effects of increasing concentrations of TECD-Fc (filled bars) or PDGF sRα (open bars) on 10 ng/ml PDGF-AA (**B**) or PDGF-AB (**D**) stimulated BrdU incorporation.

We next examined the effect of TMEFF2-ECD on PDGF stimulated proliferation. The murine fibroblast cell line NR6 expresses both PDGF receptors α and β [30], and exhibits dose-dependent proliferation in response to PDGF-AA or PDGF-AB as measured by BrdU incorporation (Fig. 5A, C). When 10 ng/ml PDGF-AA was added in the presence of increasing concentrations of Fc-tagged TECD, BrdU incorporation was inhibited in a dose-dependent manner at concentrations between 0.6 and 2,000 ng/ml of TECD-Fc (Fig. 5B). This effect was similar to that of sRα which also inhibited PDGF-AA–induced BrdU incorporation at a similar concentration range, albeit with a slightly higher efficiency. PDGF-AB–induced BrdU incorporation, on the other hand, was not affected by TECD-Fc under the same conditions (Fig. 5D). Interestingly, sRα also had little effect on PDGF-AB–induced proliferation, even though consistent with previous reports [31], PDGF-AB could bind sRαwith an affinity similar to PDGF-AA (Supplemental Fig. S1B). This may be due to the ability of PDGF-AB to bind to all 3 PDGFR dimers, αα, αβ or ββ [26], whereas PDGF-AA can signal only through PDGFR αα dimers.It is possible that PDGF-AB may have a higher affinity for the native PDGF receptor αβ dimers than for sRα, or that there may be more abundant PDGF receptor αβ dimers and/or PDGF receptor ββ dimers on these cells.

### TMEFF2 expression is downregulated in brain cancers and is negatively correlated with PDGF-A expression

**Figure 6:**
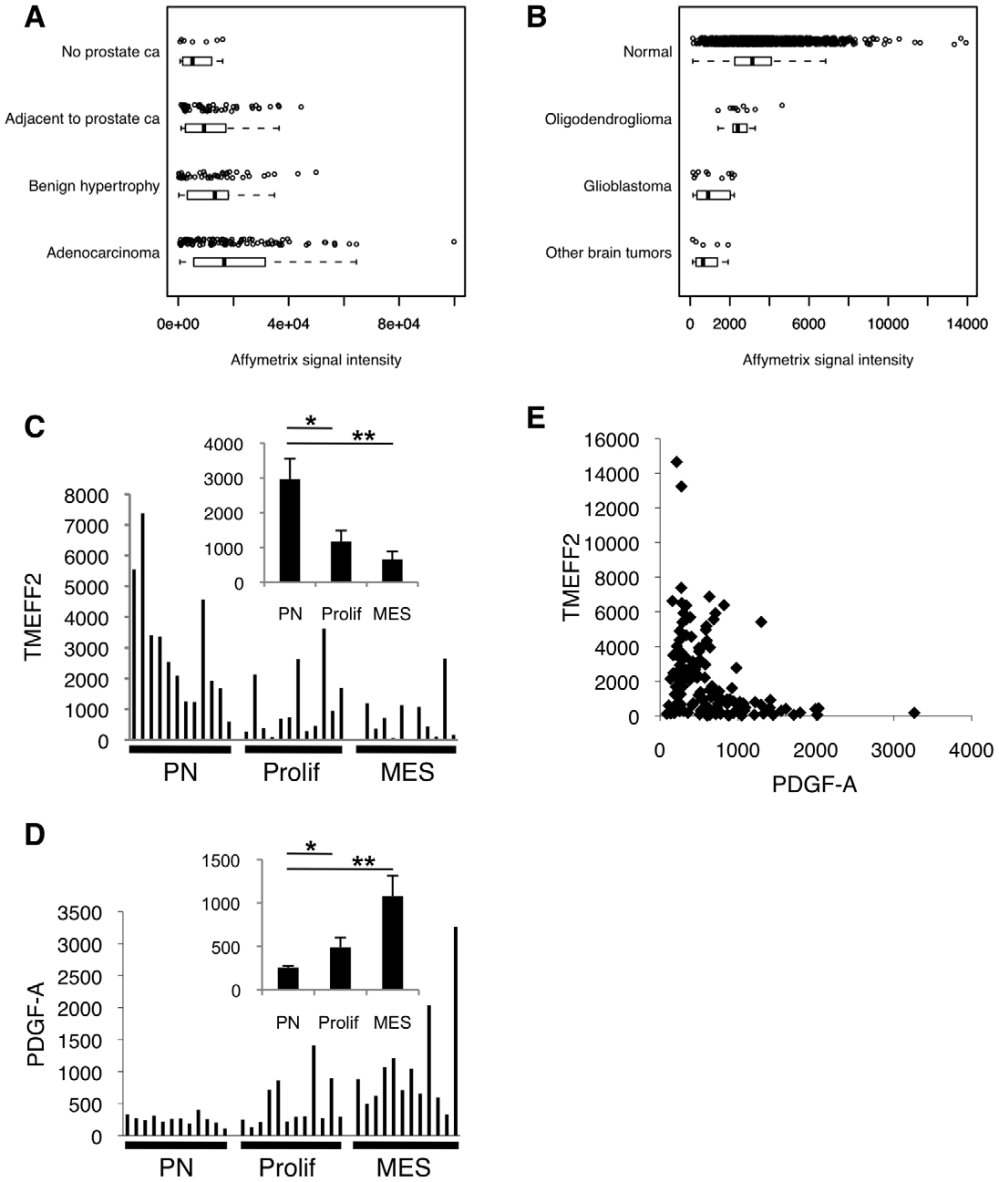
TMEFF2 expression is downregulated in glioma. (**A**) Affymetrix signal intensity of TMEFF2 expression in prostate cancer vs non-cancerous tissues based on GeneLogic data. (**B**) Affymetrix signal intensity of TMEFF2 expression in normal brain vs brain cancer tissues based on GeneLogic data. Each open circle in (**A**) & (**B**) represents one patient sample. Box-and Whisker plots are also included under the raw data to indicate the mean and the 25th and 75th percentile ranges. The whiskers are drawn at 1.5 times the interquartile range from the box. (**C**) & (**D**) Normalized signals of TMEFF2 (**C**) and PDGF-A (**D**) mRNA expression in Proneural (PN), Proliferative (Prolif), or Mesenchymal (MES) subtypes of 36 glioma samples. Mean signals for each subtype are shown as insets. * *p*≤0.05; **, *p*≤0.005. (**E**) TMEFF2 expression is negatively correlated with PDGF-A expression in 133 (76 MD Anderson and 57 UCSF) HGG samples (Pearson correlation coefficient r = −0.37). Each axis represents normalized signals of each gene. All expression data were obtained using Affymetrix HG-U133A and HG-U133B GeneChips from probe 223557_s_at for TMEFF2 and 205463_s_at for PDGF-A, respectively.

The 5′-region of TMEFF2 gene is frequently hypermethylated in some cancers [2], [3], [9]–[16], suggesting a possible tumor suppressor role for TMEFF2 in these cancers. To compare the expression levels of TMEFF2 in human tissues, we analyzed Affymetrix microarray data obtained from GeneLogic, Inc. (Gaithersburg, MD) containing multiple human tumor and normal tissue samples. Highest levels of TMEFF2 expression were found in prostate and brain tissues (Supplemental Fig. S3, S4). *In situ* hybridization experiments confirmed high levels of TMEFF2 mRNA expression in normal adult and fetal central nervous systems, as well as both malignant and non-malignant prostate tissues (Supplemental Fig. S5). The mean expression level of TMEFF2 is significantly higher in prostate cancer tissues compared to normal prostate tissues (Fig. 6A; Supplemental Fig. S3, S4), consistent with previous reports [7]. In contrast, TMEFF2 exhibits significantly lower mean levels of expression in malignant brain samples, especially in glioblastomas (GBMs), compared to normal brain tissues (Fig. 6B; Supplemental Fig. S3, S4). Most other tissues express TMEFF2 at much lower levels than brain and prostate. Several tissues also show a trend of decreased expression in cancers, such as colorectal, esophagus and stomach, with statistically significant difference in colorectal cancer samples compared to normal colon tissues (Supplemental Fig. S3, S4). These data are consistent with a possible tumor suppressor role of TMEFF2 in these tissues.

High grade gliomas (HGGs) have been classified into three molecular subtypes based on similarity to defined expression signatures: Proneural (PN), Proliferative (Prolif) and Mesenchymal (MES) [32]. The Proneural subtype expresses genes associated with normal brain and the process of neurogenesis. This subtype has been associated with a better prognosis [32], and has recently been linked to a subset of tumors exhibiting a glioma-CpG island methylator phenotype (G-CIMP) and isocitrate dehydrogenase 1 (IDH1) mutation (see below) [33]. In contrast, the other two subtypes are of poorer prognosis [32], and are characterized by a resemblance to either highly proliferative cell lines or tissues of mesenchymal origin, with gene expression programs indicative of cell proliferation or angiogenesis, respectively. Microarray analysis of TMEFF2 in a set of 36 HGG samples that included 12 prototypical cases of each subclass [32], [34] revealed significantly higher levels of TMEFF2 expression in the PN subclass than the Prolif and MES subclasses (Fig. 6C). Interestingly, PDGF-A showed an almost mirror-image, opposite trend with the highest expression in the MES subclass (Fig. 6D). Such a trend was not observed for PDGF-B in these samples (data not shown). Further analysis of microarray data in 76 HGG samples from M.D. Anderson Cancer Center (MDA) and 57 HGG samples from University of California San Francisco (UCSF) suggests a negative correlation between TMEFF2 and PDGF-A expression in both sets of samples (Fig. 6E). These data are consistent with the hypothesis that PDGF-AA may be an important growth factor required for the development of non-PN HGGs, and that TMEFF2 expression may be selected against in these HGGs that are dependent on PDGF-AA signaling.

### TMEFF2 is hypermethylated in multiple tumor types with its expression negatively correlated with methylation levels

**Figure 7:**
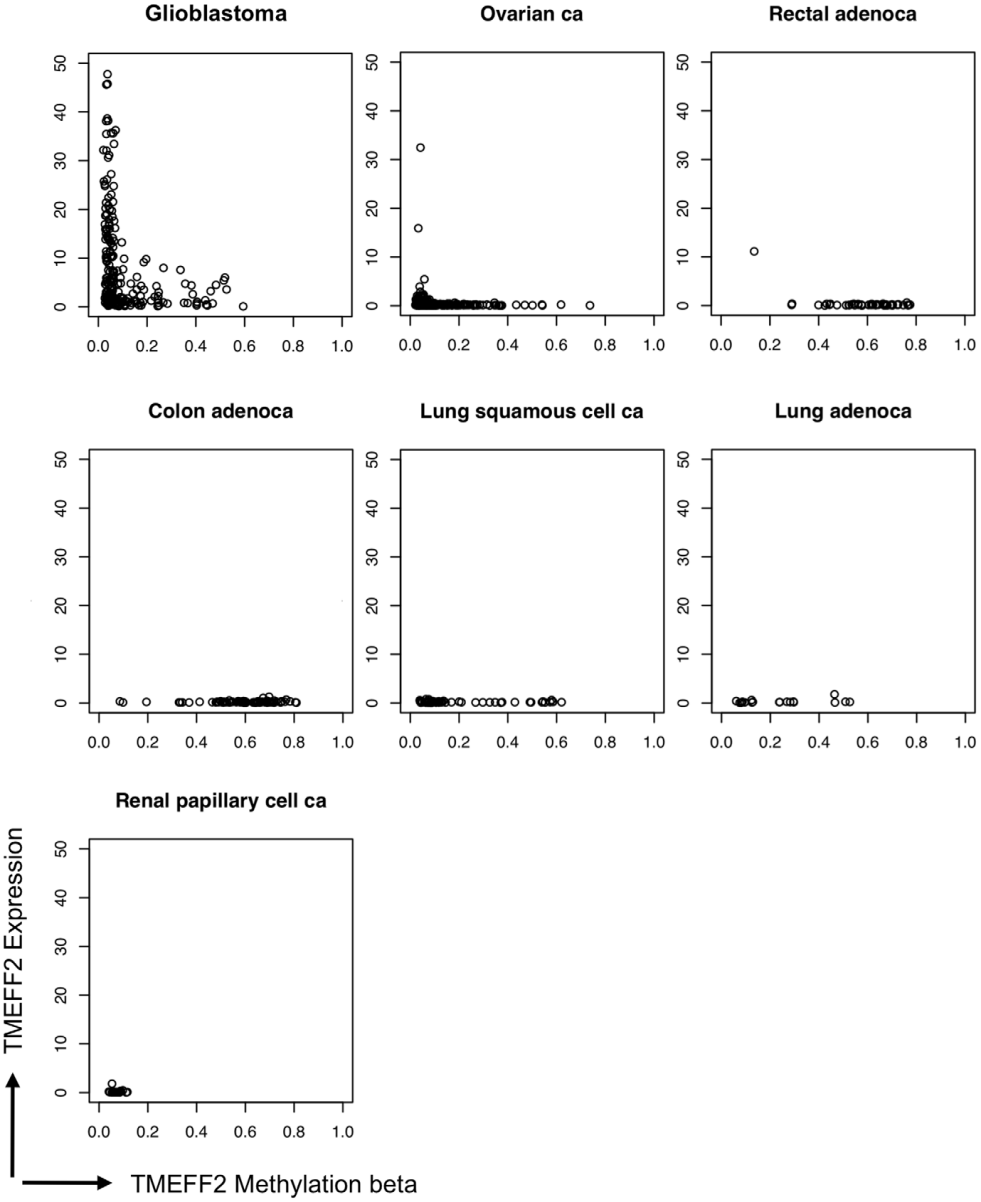
Expression vs. methylation status of TMEFF2 in 7 human tumor types. Methylation levels are plotted on the x-axis by averaging the beta values of the two Infinium probes, cg06856528 and cg18221862, and mRNA expression levels obtained on the Agilent chip are plotted on the y-axis.

Hypermethylation of the TMEFF2 gene in human cancers has been reported in several tissues including colorectal, gastric and esophageal cancers [2], [3], [9]–[12], [16]. However, these tissues express very low levels of TMEFF2 even in normal samples, making the significance of gene suppression less clear in these tumors. Since the methylation status of TMEFF2 has not been reported in glioma and most other tissues, we analyzed all publicly available data from The Cancer Genome Atlas (TCGA) with results on both Agilent expression and Infinium methylation arrays [35]. Of the seven tumor types where these data are currently available, only glioblastoma, and occasionally ovarian and rectal cancer samples show significant levels of TMEFF2 expression (Fig. 7). All samples with high levels of TMEFF2 expression correspond to low CpG island methylation states, while samples with a methylation beta value of greater than 0.1 have a suppressed expression of TMEFF2, which is especially apparent in GBM samples (t-test p-value 4×10^−14^). TMEFF2 expression is barely detectable in almost all colon adenocarcinoma, rectal adenocarcinoma, lung adenocarcinoma and lung squamous cell carcinoma samples. While the majority of these tumor samples show methylation beta values greater than 0.1, there are insufficient data available to determine whether different thresholds of methylation beta values exist in different tumor types for suppressed TMEFF2 expression, or other mechanisms exist to suppress its expression. Nevertheless, taken together with other published reports of TMEFF2 methylation in other tumor types, these data are consistent with the hypothesis that TMEFF2 is silenced through DNA methylation in a significant proportion of human cancers, including glioma and cancers of ovarian, rectal, colon and lung origins.

**Figure 8:**
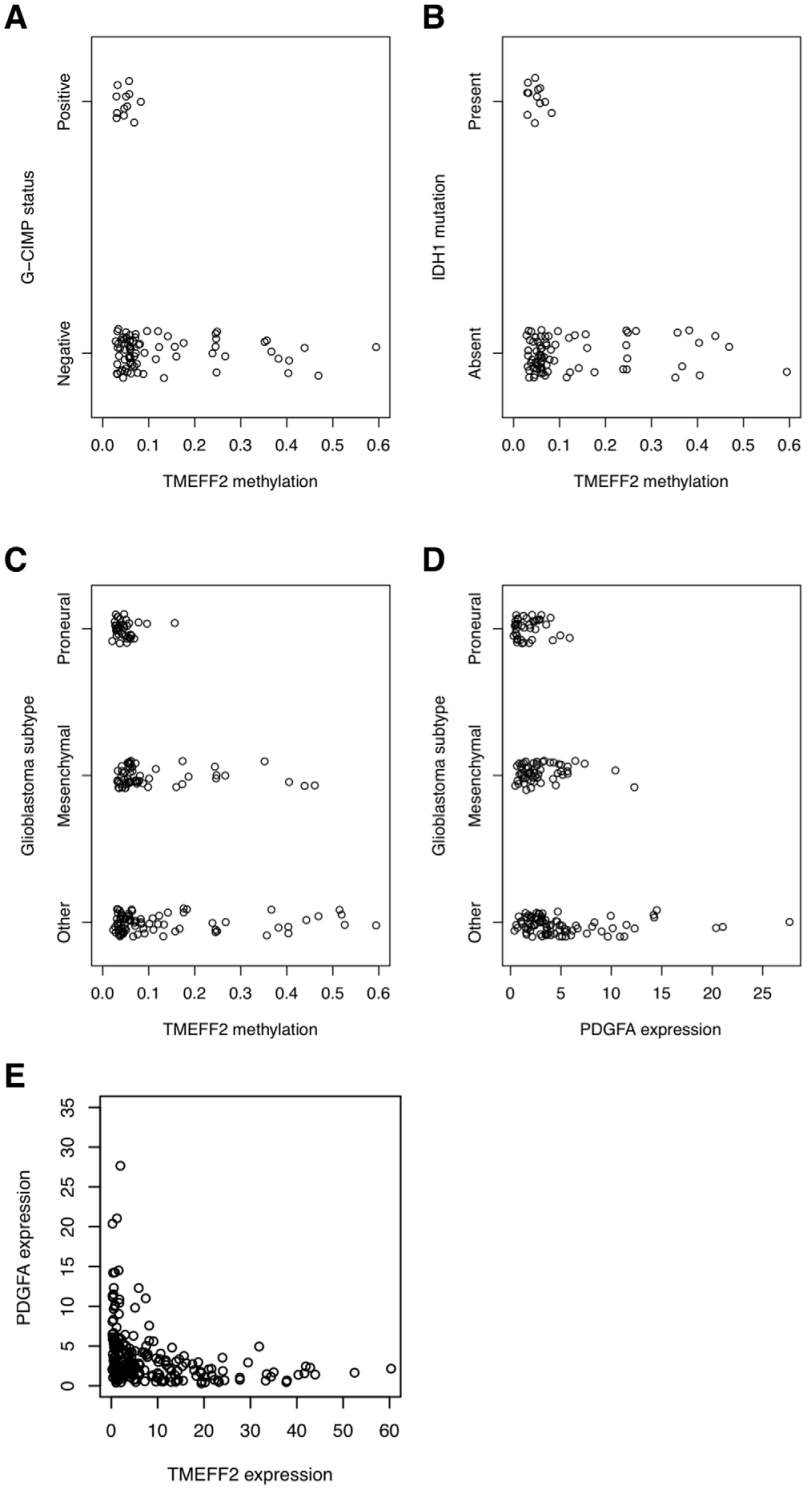
Correlates of TMEFF2 methylation and expression in TCGA glioblastoma samples. (A) TMEFF2 methylation status vs. G-CIMP status. (B) TMEFF2 methylation status vs. IDH1 mutation status. (C) TMEFF2 methylation status vs. GBM molecular subtypes. (D) PDGF-A expression vs. GBM molecular subtypes. (E) TMEFF2 expression vs. PDGF-A expression [t-test p-value = 6.6×10^−13^ between PDGF-A expression levels in samples with high TMEFF2 (expression value≥10) vs. those with low TMEFF2 (expression value<10)].

Recently, a subset of gliomas with characteristic promoter DNA methylation alterations, referred to as G-CIMP, have been identified in the context of TCGA GBM samples [33]. Interestingly, G-CIMP-positive tumors belong to a subset of Proneural tumors and are closely associated with IDH1 mutation. These tumors have a favorable prognosis within GBMs as a whole and also within the Proneural subset. To understand the relationship between TMEFF2 methylation and the G-CIMP signature, we compared the TMEFF2 methylation status against the set of TCGA GBM samples with available G-CIMP and IDH1 mutation information. Of the TCGA samples analyzed by Noushmehr et al. [33], 88 overlapped with the samples that we analyzed using the publicly available dataset. 76 of these were G-CIMP-negative and 12 were G-CIMP-positive. All 76 G-CIMP-negative samples were negative for the IDH1 mutation, while all 12 G-CIMP-positive samples were positive for the IDH1 mutation. Strikingly, tumors with a greater than 0.1 TMEFF2 methylation beta value are found exclusively within the non-G-CIMP and non-IDH1-mutant category (Fig. 8A & B). Thus, TMEFF2 does not belong to the reported G-CIMP loci; in contrast, there is a strong anti-correlation between TMEFF2 hypermethylation and G-CIMP-positive or IDH1 mutation status.

That TMEFF2 hypermethylation is not found in the G-CIMP and IDH1-mutant GBM samples is consistent with our observation that higher levels of TMEFF2 are associated with the Proneural HGGs, the subclass that the G-CIMP tumors belong to, while suppressed expression of TMEFF2 is associated with the Proliferative and Mesenchymal subclasses of HGGs (Fig. 6C). Therefore, we further analyzed the relationship between TMEFF2 methylation status and the molecular subtypes of the TCGA GBM samples. Using an unsupervised approach to classify data from TCGA, Verhaak et al. described 4 GBM transcriptomal subtypes, termed Proneural, Neural, Mesenchymal and Classical [36]. As recently reviewed in Huse et al. [37], comparison of classification schemes of Verhaak et al. and Phillips et al. [32] reveals a large degree of agreement in assignment of samples to Proneural and Mesenchymal subtypes, while the other expression subtypes are less well resolved. Therefore, we assigned “Proneural” only to those GBM samples that are classified as Proneural by both Phillips and Verhaak schemes, and “Mesenchymal” only to those classified as Mesenchymal by both schemes ([37]; C. Brennan, personal communication). All other samples are designated as “Other”. As expected, TMEFF2 methylation beta values>0.1 are almost exclusively observed in a subset of non-Proneural GBM samples, including both Mesenchymal and Other subtypes (Fig. 8C). Thus, TMEFF2 hypermethylation anti-correlates with the Proneural signature in GBMs. Consistent with the observation in HGG samples shown above, Proneural GBMs express the lowest levels of PDGF-A, compared to other GBMs (Fig. 8D). Moreover, a strong anti-correlation also exists between PDGF-A and TMEFF2 expressions in the TCGA GBM samples (Fig. 8E).

## Discussion

Follistatin domain–containing proteins have been shown to interact with growth factors or their binding partners and modulate their signaling [19], [24], [38]. For example, the follistatin domain–containing ECM–associated glycoprotein SPARC/osteonectin was reported to interact with PDGF-AB and BB (but not AA) and inhibit the binding of these ligands to their cognate receptors on fibroblasts [19]. Here we report for the first time that TMEFF2 selectively interacts with PDGF-AA via its follistatin domain–containing extracellular regions, and modulates PDGF-AA–stimulated proliferation of NR6 fibroblasts. Interestingly, both shedding of the extracellular domains of TMEFF2 [39], and a truncated splice variant of TMEFF2 encoding a secreted protein without the EGF-like and the transmembrane domains [40], have been identified in cells, suggesting a possible functional role of the extracellular region containing the follistatin domains independent of the intracellular and transmembrane regions.

First identified in a search for serum factors that stimulate the proliferation of arterial smooth muscle cells [41], PDGFs have been shown to direct a variety of cellular responses including proliferation, survival, migration, and the deposition of ECM and tissue remodeling factors (reviewed in [17] and [18]). Of the genes encoding the four PDGF ligands and their two receptor chains, mouse knockout studies have suggested that PDGF-B and PDGFRß are essential for the development of support cells in the vasculature, whereas PDGF-A and PDGFRα are more broadly required during embryogenesis, with essential roles in central nervous system, neural crest and organ development (reviewed in [18]). PDGFs have also been implicated in the etiology of human cancers. Both PDGFs and PDGFRs are upregulated in human gliomas and astrocytomas, and PDGFRα mRNA expression levels are higher in more advanced forms of gliomas than in less malignant glial tumors [42], [43]. Elevated levels of PDGF-A and PDGFRα proteins have also been observed in human prostate carcinomas [17], [44], [45]. In human gastric cancers, high levels of PDGF-A correlate with high-grade carcinomas and reduced patient survival [46]. *Pdgfra*-activating mutations have also been identified in a subset of human gastrointestinal stromal tumors [47]. Interestingly, we and others have observed highest levels of TMEFF2 expression in the central nervous system and the prostate amongst normal human tissues (Supplementary Figures S3, S4, S5 and [7]). Conversely, lower levels of TMEFF2 are found in multiple cancer tissues, especially in the malignant brain and colorectal samples, when compared to normal tissues.

The significance of the previously reported hypermethylation of TMEFF2 gene in human cancers including colorectal, gastric and esophageal cancers [2], [3], [9]–[12], [16] is confounded by the low levels of TMEFF2 expression in normal tissues of these origins. Here we report hypermethylation of TMEFF2 in several additional tumor types, including GBM, where a clear down-regulation is observed compared to high levels of TMEFF2 expression in normal brain tissues. We show that expression of TMEFF2 negatively correlates with its methylation levels in GBM and several other tumor types, further supporting a possible tumor suppressor role of TMEFF2 in these tissues. In contrast, the mean TMEFF2 mRNA expression is elevated in prostate cancer tissues, especially non-metastatic prostate cancer tissues, compared to normal prostates, suggesting a possible tissue and cell context-dependent dual function of TMEFF2 in human cancers.

We have found that TMEFF2 hypermethylation is associated with non-Proneural subtypes of GBMs, in contrast with G-CIMP methylation and IDH1 mutation status, which are associated with the Proneural subtype and lower-grade gliomas. These associations are consistent with our finding of higher levels of TMEFF2 expression in the Proneural subtype. Moreover, we observe an exclusivity relationship between TMEFF2 hypermethylation and G-CIMP methylation, in that none of our samples show both types of methylation patterns. These data suggest that TMEFF2 is preferentially hypermethylated and suppressed in a subset of non-Proneural and non-G-CIMP HGGs, and that TMEFF2 methylation may be associated with worse prognosis.

We also observed an anti-correlation between TMEFF2 expression and PDGF-A expression in the GBM and HGG samples, with lowest levels of PDGF-A expression observed in the Proneural subtype compared to other subtypes. Interestingly, despite the high levels of TMEFF2 and low levels of PDGF-A expression, PDGFRα amplification appears to be associated with the Proneural signature of GBM, which may also display elevated PDGF signaling signature through increased PDGF-B protein levels and elevated phosphorylation of PDGFRβ [36], [48]. In fact, a broad range of human gliomas display altered PDGF pathway activity, strongly suggesting that this signaling axis plays central roles in the events underlying gliomagenesis [49]. It is possible that TMEFF2 serves as a tumor suppressor in normal brain by inhibiting signaling via PDGF-AA. Hypermethylation and downregulation of TMEFF2 may facilitate tumorigenesis in the tumors that express high levels of PDGF-A by releasing this inhibition. This mechanism of tumorigenesis can only function when PDGF-AA is present and may select for both low TMEFF2 and high PDGF-A expression. Of note, Verhaak et al. reported PDGF-A overexpression as one of the gene signatures in the “Classical” subtype of GBMs [36]; this subtype also exhibited the highest proportion of samples with TMEFF2 hypermethylation in our analysis (Supplemental Fig. S7). In contrast, Proneural and other tumors with low PDGF-A expression may utilize or be selected for a different mechanism to activate PDGF signaling despite the low levels of PDGF-A expression, such as upregulation of PDGF-B [48] or amplification of PDGFR [36], without the repression of TMEFF2. It should be noted that PDGFRα can be activated by ligands other than PDGF-AA, such as PDGF-BB and PDGF-CC, therefore can signal in the absence of PDGF-A.

Our findings not only suggest a connection between the role of TMEFF2 in PDGF signaling and the potential tumor suppressor function of TMEFF2, but also provide possible explanations for the seemingly conflicting roles of TMEFF2 in human cancers. It was previously reported that soluble forms of TMEFF2 extracellular domain could weakly stimulate erbB-4/HER4 tyrosine phosphorylation in MKN 28 gastric cancer cells [1], and promote survival of mesencephalic dopaminergic neurons in primary culture [6]. Although we did not detect a direct interaction between the EGF domain of TMEFF2 and HER4, it is conceivable that the EGF-like domain might have growth factor-like functions opposite to its follistatin domains. Alternatively, the interaction between TMEFF2 and PDGF-AA may either function to sequester the active PDGF ligand away from its receptor, or act as a carrier to concentrate or stabilize the PDGF ligand, depending on the local concentrations of these proteins in different cellular contexts.

## Materials and Methods

### Cell culture and reagents

The HEK 293 (Genentech, [50]) and NR6 cell lines [51] were maintained at 37°C and 5% CO_2_ in DMEM/Ham's F-12 (1∶1) containing 10% fetal bovine serum (FBS) and RPMI 1640 containing 10% calf serum, respectively. Recombinant human PDGF-AA, AB, BB, CC and DD, recombinant human PDGF receptor α extracellular domain (PDGF sRα), recombinant human PDGFRβ-Fc, goat anti-human PDGF, and biotinylated goat anti-human PDGF-A and PDGF-B antibodies were obtained from R&D Systems (Minneapolis, MN). Rabbit anti-PDGF-A polyclonal antibody was obtained from Santa Cruz Biotechnology (Santa Cruz, CA). Mouse anti-FLAG antibody was obtained from Sigma (St. Louis, MO). Other recombinant proteins and antibodies were generated at Genentech.

### Generation of the various deletion and fusion TMEFF2 constructs

The full-length TMEFF2 open reading frame (GenBank Accession No. NM_016192) was cloned into a modified pRK vector containing a CMV promoter. The FLAG-tagged extracellular domain of TMEFF2 (TECD) was cloned into the same vector by PCR amplification using forward primer 5′- CTATCGATCTATCGATATGGTGCTGTGGGAGT-3′ and reverse primer 5′-GACTCTAGAGTCACTTGTCATCGTCGTCCTTGTAGTCGGCGCGCCACTTTTTTTCACAGTGTT-3′ with the FLAG tag (amino acid sequence WRADYKDDDDK) fused in-frame to the CT of the end of the EGF domain. TECD-Fc was generated similarly using the same forward primer and reverse primer 5′-CTGGGCGCGCCACTTTTTTTCACAGTGTT-3′ and cloned into the same vector containing the human Fcγ sequence which was fused in-frame 3′ to the end of the EGF domain. The gD-tagged full-length TMEFF2 was cloned into the same vector with a 5′ gD tag (amino acid sequence KYALADASLKMADPNRFRGKDLPVLSGR) attached in-frame to the predicted start of the mature protein. gD-TMEFF2-ΔFS I and TMEFF2-ΔFS I/II were PRC amplified with the same reverse primer 5′-CGACTCTAGATTAGATTAACCTCGTGGACGCT-3′ and either 5′-CTGCTCGAGTGTGATATTTGCCAGTTTGGTG-3′ or 5′-CTGCTCGAGACACCACATACCTTGTCCGGAAC-3′ as forward primer, respectively.

### ELISA to measure binding between TMEFF2, PDGF and other proteins

For the TMEFF2 coat format, MaxiSorp 96-well microwell plates (Thermo Scientific Nunc, Roskilde, Denmark) were coated with 1 µg/ml TECD-FLAG (Genentech) in 50 mM carbonate buffer, pH 9.6, overnight at 4°C. Plates were washed with PBS, pH 7.4, containing 0.05% polysorbate 20 and blocked with 0.5% bovine serum albumin, 15 parts per million Proclin 300, in phosphate buffered saline (PBS), pH 7.4 for 1 hour at room temperature. Serially diluted PDGF-AA, PDGF-AB, PDGF-BB or Fc-fusion proteins in PBS containing 0.5% BSA, 0.05% polysorbate 20, and 15 parts per million Proclin 300 were added to the plates and incubated for 2 hours. Bound PDGF was detected by adding biotinylated goat anti-human PDGF-AA, PDGF-AB or PDGF-BB to the plates and incubating for one hour, followed by adding horseradish peroxidase (HRP) conjugated streptavidin (GE Healthcare, Piscataway, NJ) and incubating for 30 min, with a wash step in between. Bound Fc-fusion protein was detected by adding goat anti-human Fc-HRP (Jackson ImmunoResearch, West Grove, PA). After a final wash, the substrate 3,3′,5,5′-tetramethyl benzidine (Kirkegaard & Perry Laboratories) was added. The reaction was stopped by adding 1 M phosphoric acid and absorbance was read at 450 nm on a Multiskan Ascent reader (Thermo Scientific, Hudson, NH). The titration curves were fitted using a four-parameter nonlinear regression curve-fitting program (KaleidaGraph, Synergy software, Reading, PA).

For the PDGF coat format, plates were coated with 1 µg/ml PDGF-AA, PDGF-AB, PDGF-BB, PDGF-CC, or PDGF-DD. Serially diluted TECD-Fc (Genentech) or other Fc-fusion proteins were added to the plates. Bound protein was detected using goat anti-human Fc-HRP (Jackson ImmunoResearch, West Grove, PA).

### ELISA to measure binding of PDGF receptor α to PDGF

To measure binding of soluble PDGF receptor α to PDGF, recombinant human PDGF receptor α extracellular domain (PDGF sRα) was biotinylated using biotin-X-NHS (Research Organics, Cleveland, OH). Serially diluted biotinylated human recombinant PDGF sRα was added to PDGF-AA, PDGF-AB or PDGF-CC coated wells. Bound receptor was detected using streptavidin-HRP.

To measure blocking of TECD-Fc binding to PDGF-AA by PDGF sRα, serially diluted PDGF sRα was pre-mixed with TECD-Fc (final concentration 70 ng/ml) and added to the PDGF-AA coated plate. Bound TECD-Fc was detected using goat anti-human Fc-HRP.

### Immunoprecipitation and Western blot

For binding of PDGF ligands to membrane-bound TMEFF2 proteins, 293 cells were transfected with the various TMEFF2 constructs and changed to fresh growth medium containing 5 µg/ml PDGF-AA or AB 48 hours after transfection. After 30 minutes of incubation unbound PDGF ligands were washed away with ice cold PBS and cells were lysed in lysis buffer containing 50 mM Tris pH 8.0, 150 mM NaCl, 1 mM EDTA, 1% NP-40, protease and phosphatase inhibitors, pre-cleared with protein G sepharose, and immunoprecipitated with anti-TMEFF2, anti-PDGF, or anti-gD antibodies. The immune complexes were dissociated with SDS sample buffer with β-mercaptoethanol and resolved by 4–20% Tris-Glycine SDS PAGE, transferred to nitrocellulose membranes, and detected with the indicated antibodies using enhanced chemiluminescence.

### NR6 proliferation assays

The NR6 proliferation assay was carried out using a 5-Bromo-2′-deoxy-uridine (BrdU) labeling and detection kit (Roche). The indicated concentrations of PDGF-AA or AB were added to quiescent confluent cultures of NR6 cells in RPMI 1640 supplemented with 1× Serum Replacement 1 (Sigma) on 96-well microplates, either alone or after pre-mixing with increasing concentrations of TECD-Fc or sRα for 1 hour at 37°C. After 18 hours at 37°C and 5% CO_2_, BrdU labeling solution was added to each well and the subsequent labeling and detection were carried out following the manufacturer's protocols. BrdU incorporation was measured as absorbance at 405 nm with a reference wavelength at 490 nm.

### Microarray analysis

Gene expression profiling and analysis of microarray data were performed as previously reported [32], [52] using probe 223557_s_at for TMEFF2 and 205463_s_at for PDGF-A, respectively. Signal intensity values from Microarray Analysis Suite version 5 were utilized with a scaling factor of 500 for all analysis of microarray data. The raw Affymetrix data for TMEFF2 in the GeneLogic tissues are given in Table S1. The microarray data for HGG samples have been submitted to Gene Expression Omnibus (GEO), and the accession number for the data series is GSE4271 [32]. All data are MIAME compliant.

### Methylation and expression analysis of TCGA data

We obtained data from the Cancer Genome Atlas (TCGA) that was publicly available as of July 29, 2010, on both the Illumina Infinium methylation microarray and the Agilent G4502A expression microarray. We correlated samples based on the MAGE tables provided and found dual methylation and expression measurements for 86 colon adenocarcinomas, 226 glioblastoma samples, 36 renal papillary cell carcinomas, 21 lung adenocarcinomas, 69 lung squamous cell carcinomas, 535 ovarian carcinomas, and 53 rectal adenocarcinomas.

Methylation was measured using the beta value taken from the Level 2 files provided by TCGA. From the TCGA array description files, we identified two CpG site methylation probes for TMEFF2: cg06856528 and cg18221862. These probe sequences are located at (−204 to −155) and (−29 to +20) relative to the translation start codon, within a CpG island described previously [3]. We found correlations between the beta values for these probes to be above 0.80 for the colon adenocarcinoma, lung adenocarcinoma, and lung squamous cell carcinoma data sets, but 0.73 for gliomas, 0.67 for rectal adenocarcinomas, 0.62 for ovarian carcinomas, and 0.25 for renal papillary cell carcinomas (Supplemental Fig. S6A). We used the average of the two beta values as our estimate for methylation levels.

Expression was measured using the antilog of the log2 lowest normalized values from the Level 2 files provided by TCGA. The array description files showed three probes belonging to TMEFF2: A_23_P125382, A_23_P125383, and A_23_P125387. Pairwise correlations among these expression values were 0.94–0.95 (Supplemental Fig. S6B). We used the average of these probe values as our estimate for expression levels. The probe A_23_P113701 was used for PDGF-A expression.

TCGA samples having the same identifier as those reported by Noushmehr et al [33] were used for comparison between TMEFF2 methylation levels and their G-CIMP and IDH1 status. The subtype classifications of TCGA GBM samples according to either Phillips et al. or Verhaak et al. have been reported in summary (Huse et al., 2011 [37]) and individual sample classifications were kindly provided by Dr. Cameron Brennan. Tumors classified as “Proneural” or “Mesenchymal” by both signatures are assigned these two subtypes in Figures 8C and 8D, and all other samples are classified as “Other”.

## Supporting Information

Table S1 Affymetrix signal intensity of TMEFF2 from GeneLogic tissues with probe 223557_s_at on HG-U133A and HG-U133B GeneChips.
(XLS)

Figure S1 TECD-Fc selectively interacts with PDGF-AA. (**A**) PDGF-AA, but not AB, BB, CC or DD, binds to TECD-Fc. TECD-Fc was applied to wells coated with recombinant human PDGF-AA, AB, BB, CC or DD and detected with HRP-conjugated anti-human Fcγ. (**B**) sRα binds to all three recombinant human PDGFs: AA, AB and BB. Biotinylated recombinant sRα (sRα-bio) was applied to wells coated with recombinant human PDGF-AA, AB or BB and detected with streptavidin-HRP. (**C**) 70 ng/ml TECD-Fc was mixed with increasing concentrations of sRα and applied to PDGF-AA coated wells. Binding between TECD-Fc and PDGF-AA was detected using goat anti-human Fc-HRP.
(TIF)

Figure S2 gD-tagged TMEFF2 proteins are expressed on the cell surface as detected by an anti-gD antibody. FACS analysis of 293 cells expressing the gD-tagged full-length TMEFF2 or deletion mutants lacking either FS I or both FS modules using anti-gD mAb (black) and four mAbs (red, green, orange and blue) recognizing the FS I module of TMEFF2. Biotinylated anti-mouse IgG was used as a secondary reagent followed by streptavidin-PE. Filled purple, no primary antibody control.
(TIF)

Figure S3 Comparative transcript expression profiles of TMEFF2 in human tissues based on GeneLogic data. The mRNA expression patterns for TMEFF2 across thousands of human cancer (red) and normal (green) tissue specimens using probe 223557_s_at on chips HG-U133A and B are shown.
(TIF)

Figure S4 TMEFF2 expression is down-regulated in some cancers. (**A**) Bar-graphs of mean TMEFF2 mRNA expression levels in indicated tissues based on GeneLogic data. Error bars represent standard errors of the mean. (**B**) Number of tissues analyzed in each category. [N], Normal tissues; [C], Cancer tissues; [M], metastatic tissues; * *p*<0.05 and ** *p*<0.005 compared to normal.
(TIF)

Figure S5 *In situ* hybridization (ISH) analysis of TMEFF2 mRNA expression in normal adult brain and cerebellum (**A**), fetal spinal cord and spinal ganglion (**B**), non-malignant prostate (**C**) and prostate cancer tissues collected on tissue microarrays (TMA) (**D**). Upper panels, H & E stains; lower panels, ISH signals (white).
(TIF)

Figure S6 (**A**) Correlations between the beta values of two TCGA array methylation probes for TMEFF2 in the tissues analyzed: colon adenocarcinoma (coad), lung adenocarcinoma (luad), lung squamous cell carcinoma (lusc), glioma (gbm), rectal adenocarcinoma (read), ovarian carcinoma (ov), and renal papillary cell carcinoma (kirp). (**B**) Pairwise correlations among the three expression probes belonging to TMEFF2.
(TIF)

Figure S7 TMEFF2 methylation (**A**) vs. PDGF-A expression (**B**) in GBM subtypes. Each GBM sample is classified according their classification by both Verhaak and Phillips schemes (denoted as Verhaak scheme:Phillips scheme).
(TIF)

Figure S8 (**A**) Efficiency of anti-TMEFF2 immunoprecipitation of full-length or intracellular domain–truncated TMEFF2 expressed on 293 cells compared to inputs in the whole cell lysates (WCL). (**B**) Efficiency of PDGF-A co-immunoprecipitation with full-length TMEFF2 with or without a gD tag compared to 5 ng of recombinant PDGF-AB or the amount of surface-bound PDGF-A in the whole cell lysates (WCL).
(TIF)
