# Supplementary figures and images for: TMEFF2 Is a PDGF-AA Binding Protein with Methylation-Associated Gene Silencing in Multiple Cancer Types Including Glioma
Source: PLoS One. 2011 Apr 29;6(4):e18608. doi: 10.1371/journal.pone.0018608 (PMC3084709; doi:10.1371/journal.pone.0018608)

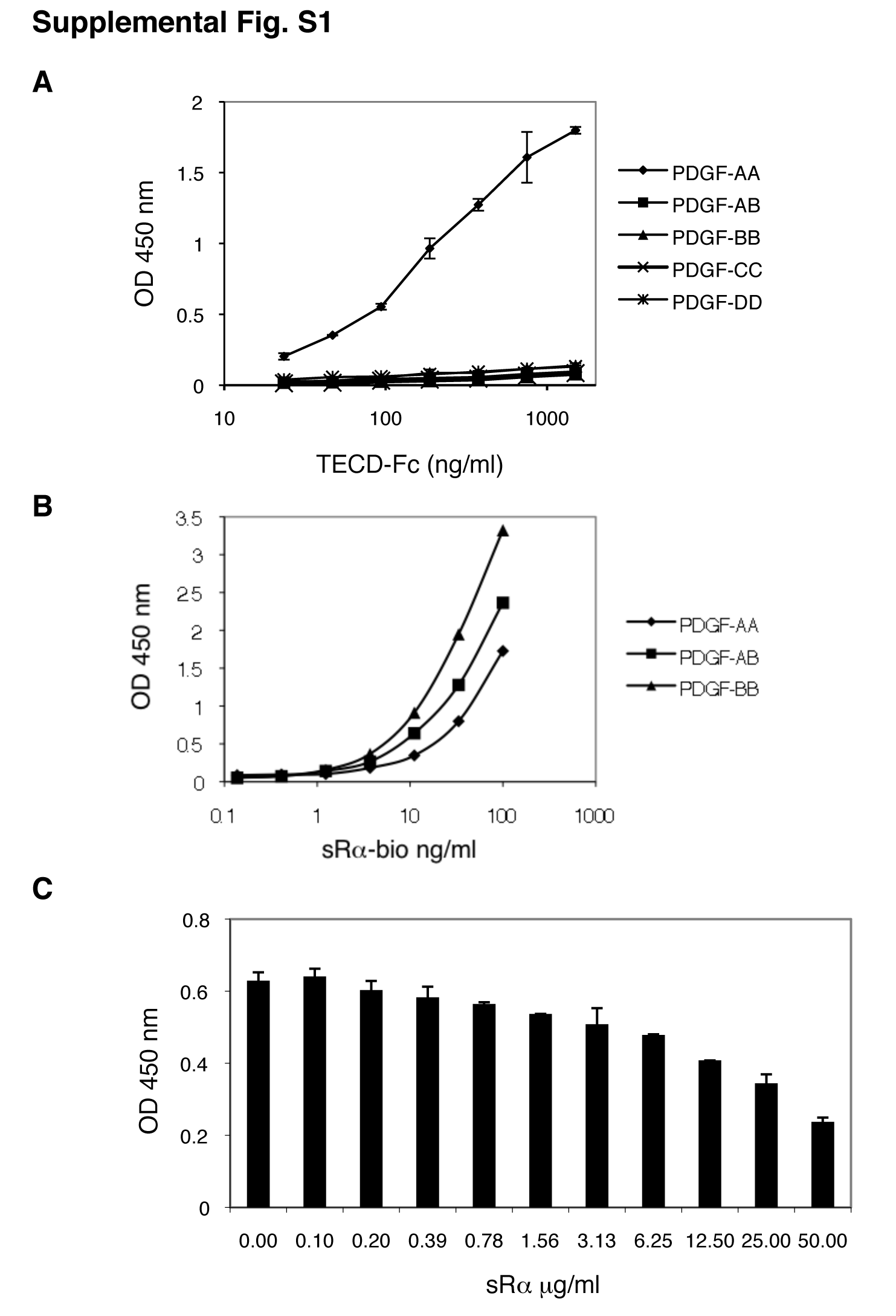

Supplement: Figure S1 — TECD-Fc selectively interacts with PDGF-AA. (A) PDGF-AA, but not AB, BB, CC or DD, binds to TECD-Fc. TECD-Fc was applied to wells coated with recombinant human PDGF-AA, AB, BB, CC or DD and detected with HRP-conjugated anti-human Fcγ. (B) sRα binds to all three recombinant human PDGFs: AA, AB and BB. Biotinylated recombinant sRα (sRα-bio) was applied to wells coated with recombinant human PDGF-AA, AB or BB and detected with streptavidin-HRP. (C) 70 ng/ml TECD-Fc was mixed with increasing concentrations of sRα and applied to PDGF-AA coated wells. Binding between TECD-Fc and PDGF-AA was detected using goat anti-human Fc-HRP. (TIF) [file pone.0018608.s002.tif]

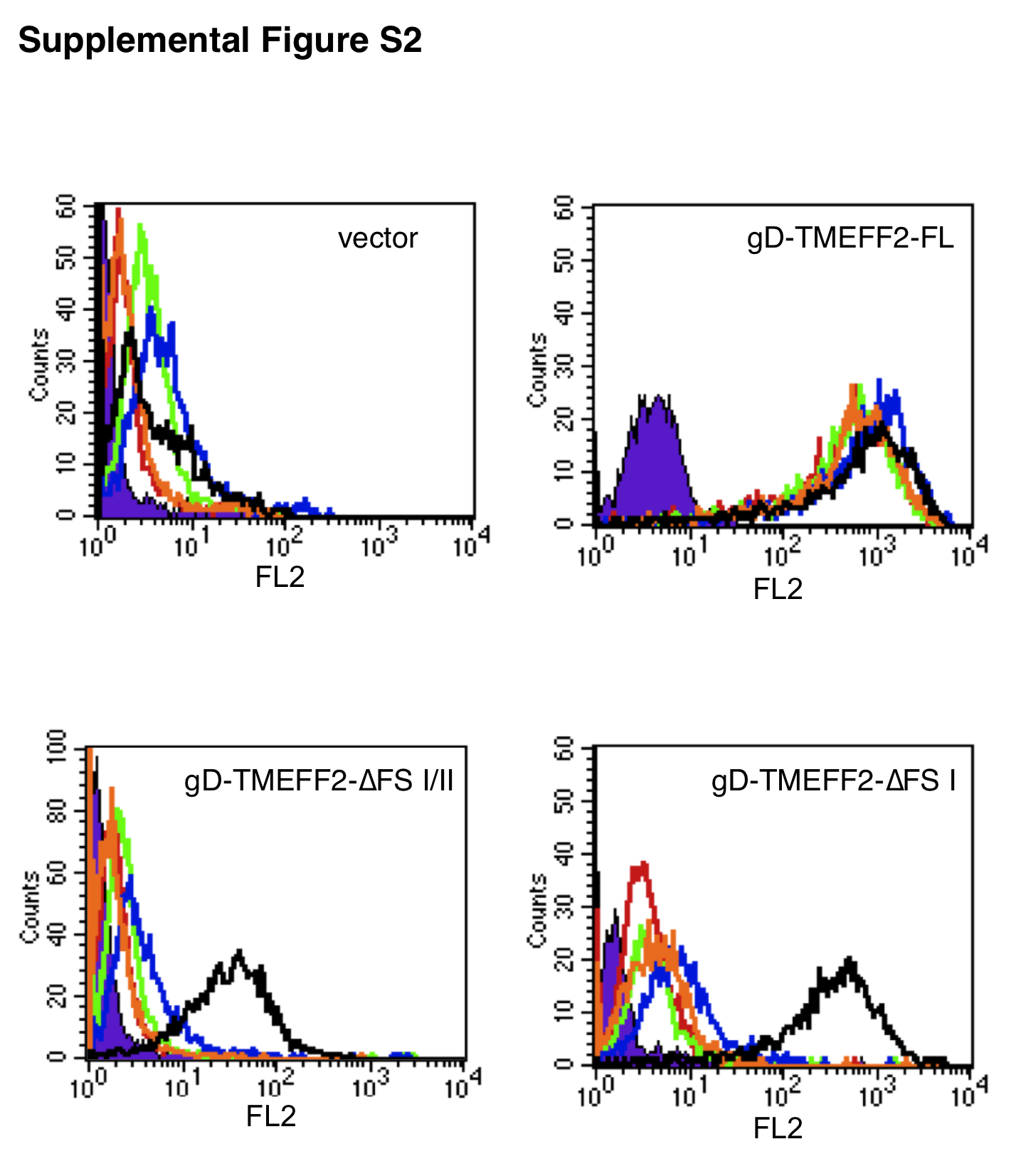

Supplement: Figure S2 — gD-tagged TMEFF2 proteins are expressed on the cell surface as detected by an anti-gD antibody. FACS analysis of 293 cells expressing the gD-tagged full-length TMEFF2 or deletion mutants lacking either FS I or both FS modules using anti-gD mAb (black) and four mAbs (red, green, orange and blue) recognizing the FS I module of TMEFF2. Biotinylated anti-mouse IgG was used as a secondary reagent followed by streptavidin-PE. Filled purple, no primary antibody control. (TIF) [file pone.0018608.s003.tif]

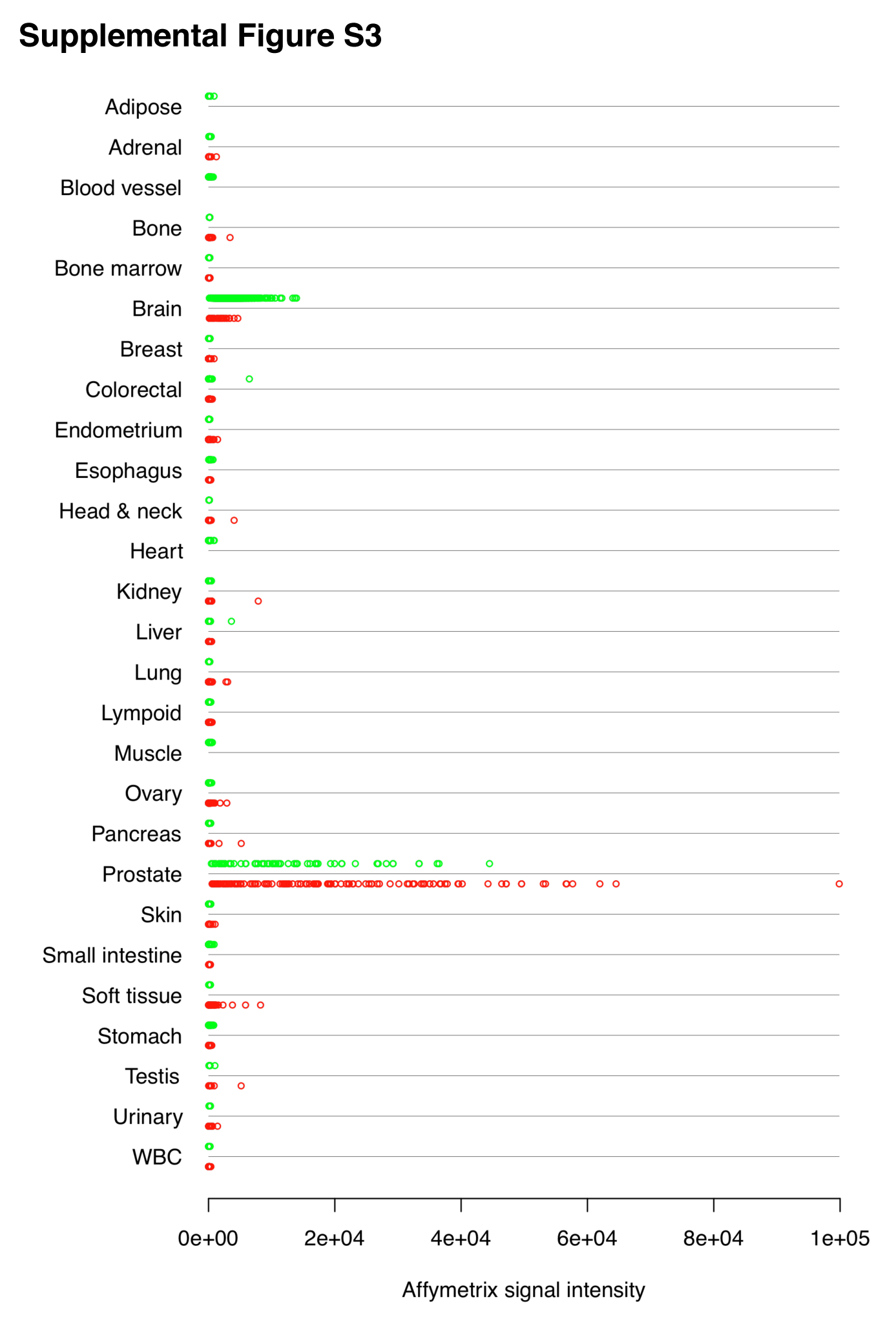

Supplement: Figure S3 — Comparative transcript expression profiles of TMEFF2 in human tissues based on GeneLogic data. The mRNA expression patterns for TMEFF2 across thousands of human cancer (red) and normal (green) tissue specimens using probe 223557_s_at on chips HG-U133A and B are shown. (TIF) [file pone.0018608.s004.tif]

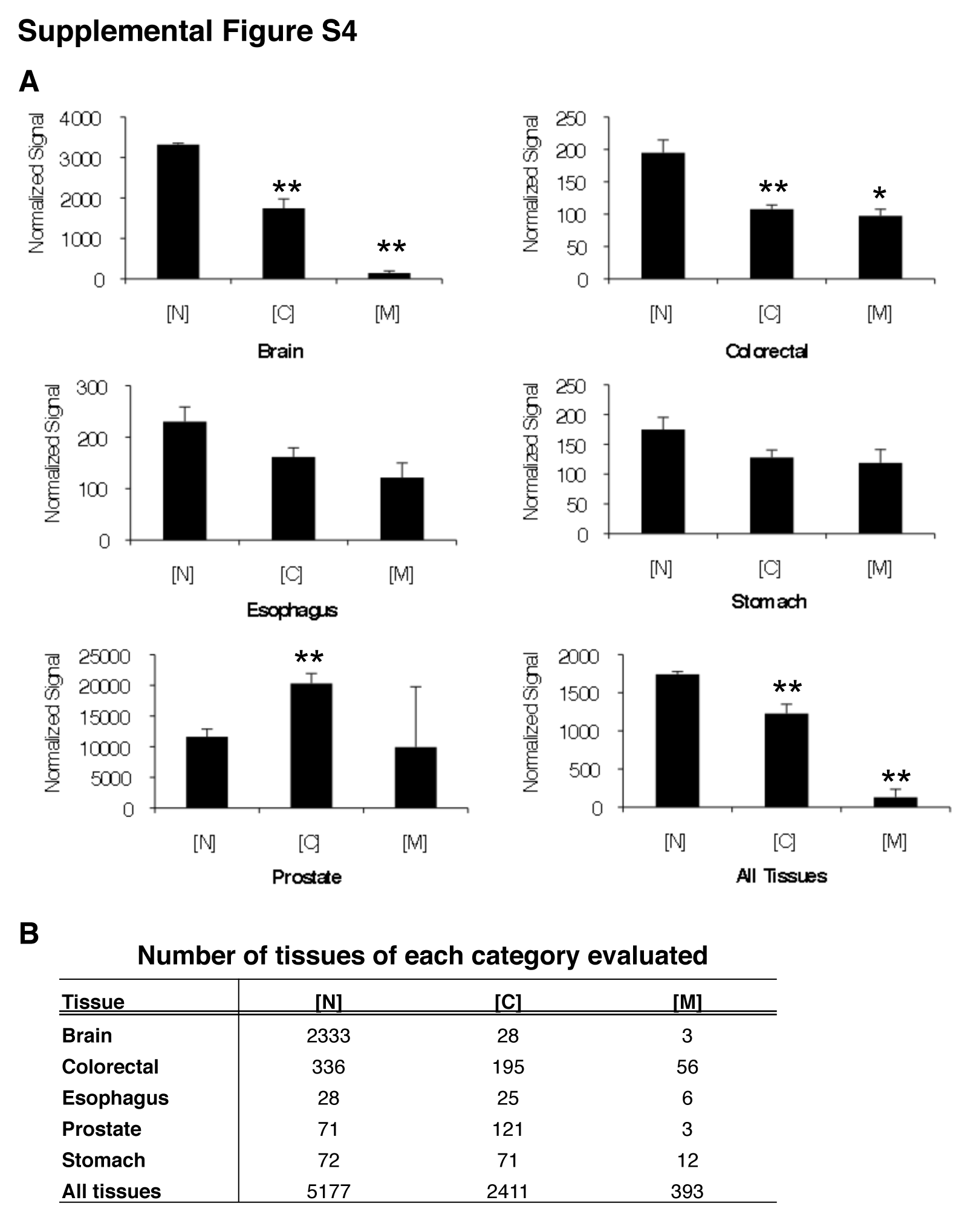

Supplement: Figure S4 — TMEFF2 expression is down-regulated in some cancers. (A) Bar-graphs of mean TMEFF2 mRNA expression levels in indicated tissues based on GeneLogic data. Error bars represent standard errors of the mean. (B) Number of tissues analyzed in each category. [N], Normal tissues; [C], Cancer tissues; [M], metastatic tissues; * p<0.05 and ** p<0.005 compared to normal. (TIF) [file pone.0018608.s005.tif]

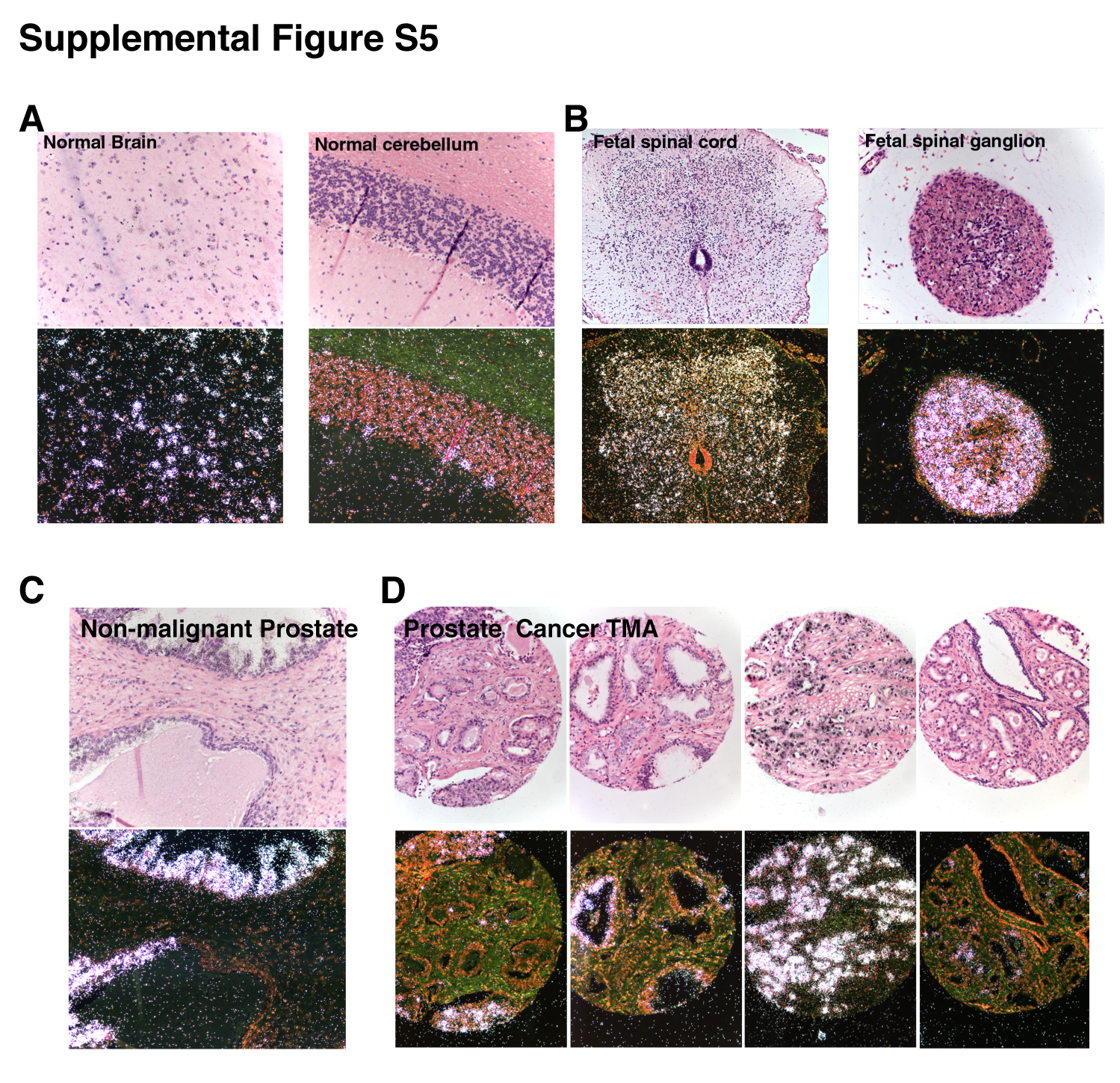

Supplement: Figure S5 — In situ hybridization (ISH) analysis of TMEFF2 mRNA expression in normal adult brain and cerebellum (A), fetal spinal cord and spinal ganglion (B), non-malignant prostate (C) and prostate cancer tissues collected on tissue microarrays (TMA) (D). Upper panels, H & E stains; lower panels, ISH signals (white). (TIF) [file pone.0018608.s006.tif]

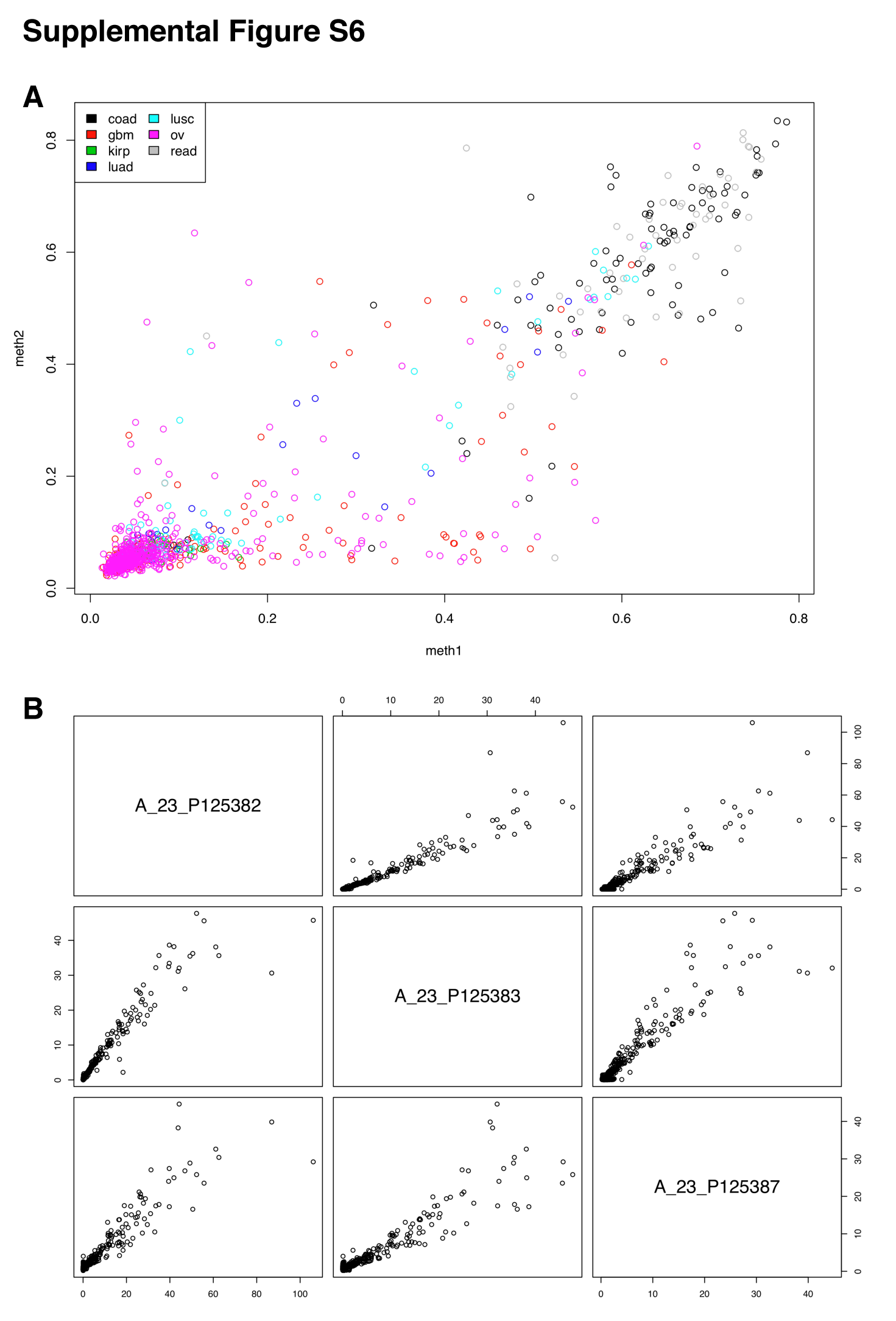

Supplement: Figure S6 — (A) Correlations between the beta values of two TCGA array methylation probes for TMEFF2 in the tissues analyzed: colon adenocarcinoma (coad), lung adenocarcinoma (luad), lung squamous cell carcinoma (lusc), glioma (gbm), rectal adenocarcinoma (read), ovarian carcinoma (ov), and renal papillary cell carcinoma (kirp). (B) Pairwise correlations among the three expression probes belonging to TMEFF2. (TIF) [file pone.0018608.s007.tif]

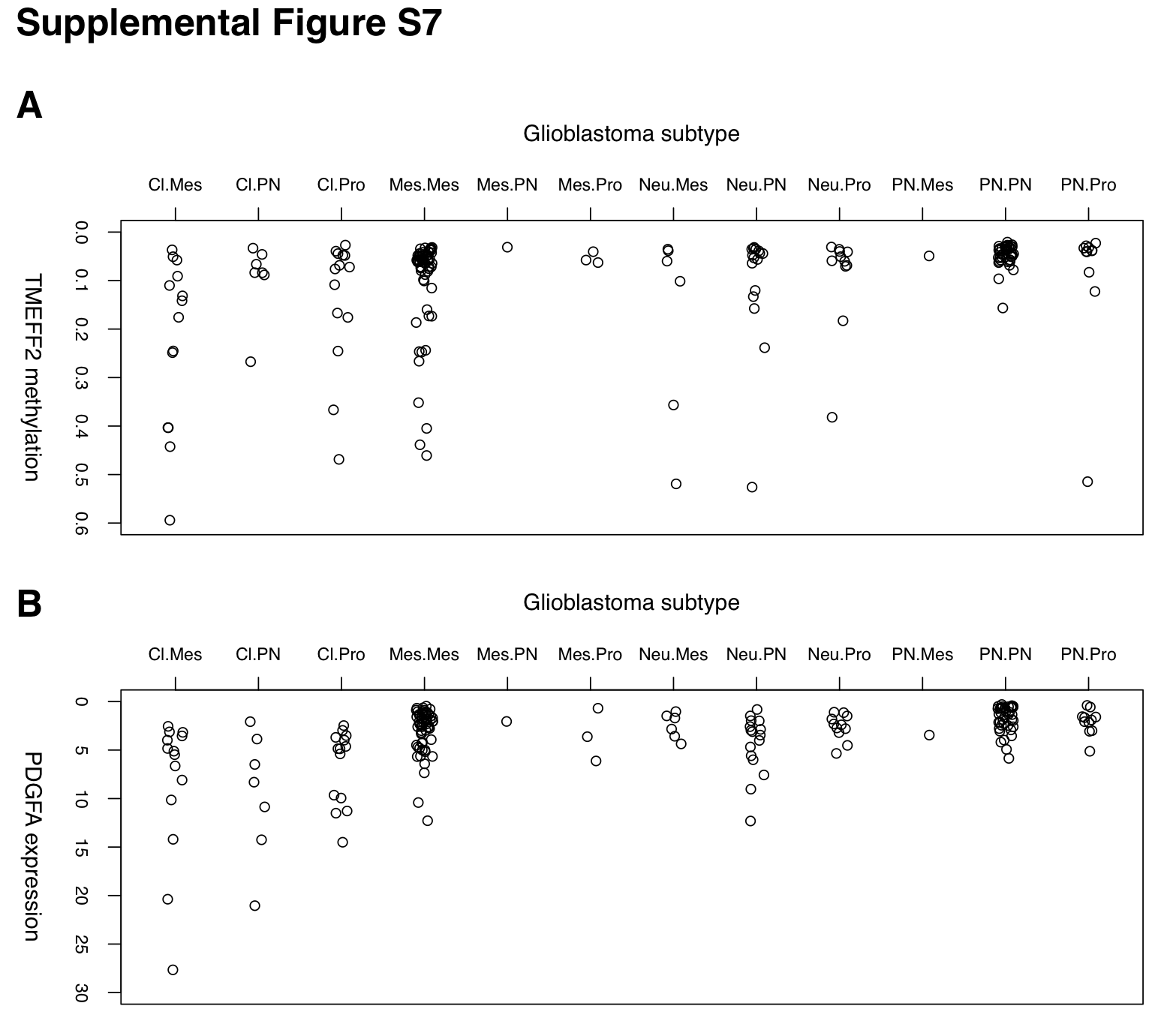

Supplement: Figure S7 — TMEFF2 methylation (A) vs. PDGF-A expression (B) in GBM subtypes. Each GBM sample is classified according their classification by both Verhaak and Phillips schemes (denoted as Verhaak scheme:Phillips scheme). (TIF) [file pone.0018608.s008.tif]

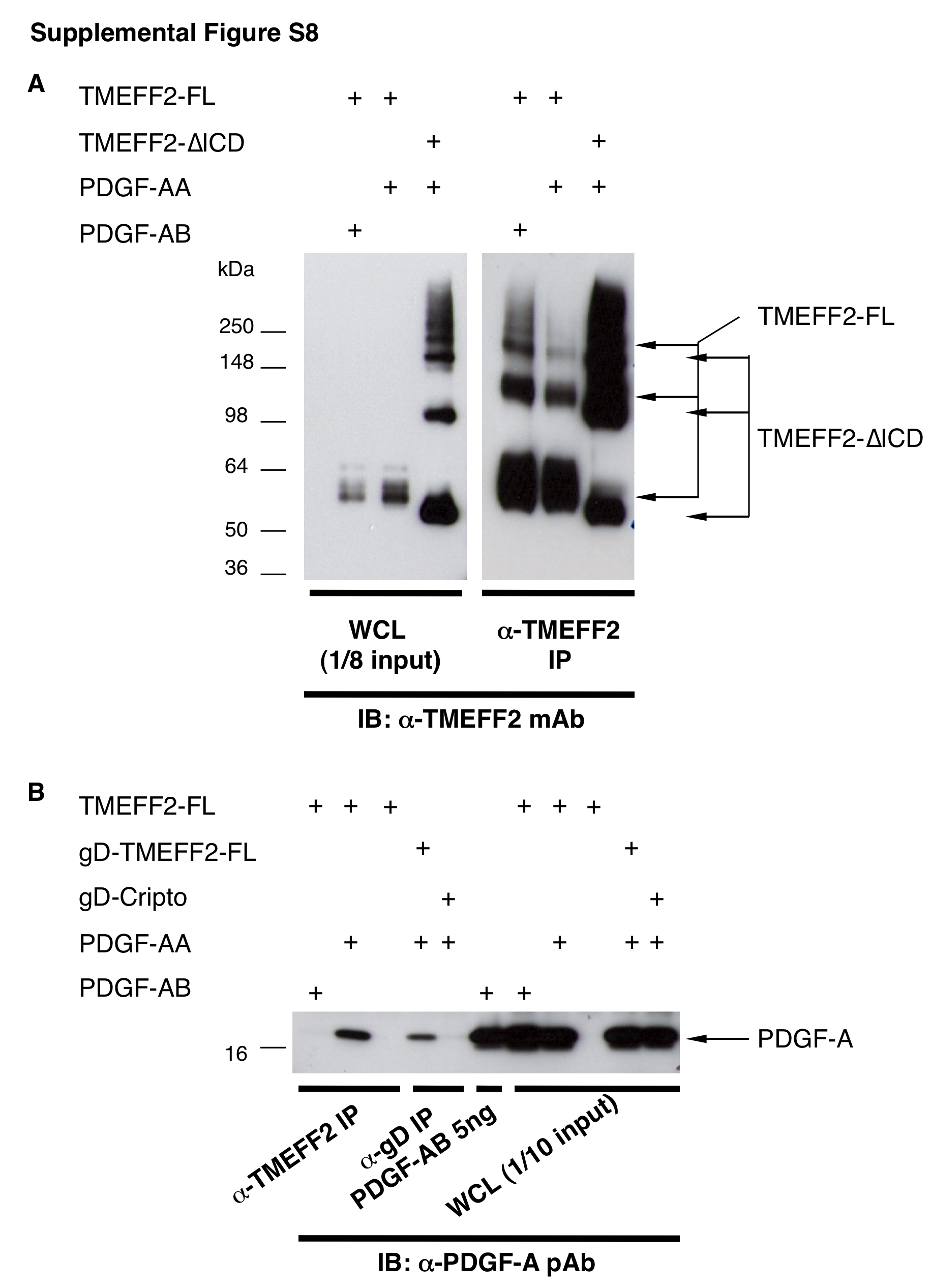

Supplement: Figure S8 — (A) Efficiency of anti-TMEFF2 immunoprecipitation of full-length or intracellular domain–truncated TMEFF2 expressed on 293 cells compared to inputs in the whole cell lysates (WCL). (B) Efficiency of PDGF-A co-immunoprecipitation with full-length TMEFF2 with or without a gD tag compared to 5 ng of recombinant PDGF-AB or the amount of surface-bound PDGF-A in the whole cell lysates (WCL). (TIF) [file pone.0018608.s009.tif]
